# Two Remarkable Cases of Intestinal Perforation

**Published:** 1905-12

**Authors:** Emory Lanphear

**Affiliations:** St. Louis, Mo.; Formerly Professor of Operative Surgery in the Kansas City Medical College and Professor of Surgery in the St. Louis College of Physicians and Surgeons


For Texas Medical Journal.

### Two Remarkable Cases of Intestinal Perforation.

BY EMORY LANPHEAR, M. D., PH. D., LL. D., ST. LOUIS, MO.

Formerly Professor of Operative Surgery in the Kansas City Medical College and Professor of Surgery in the St. Louis College of Physicians and Surgeons.

E. S., male, age 39; patient of Dr. Emory Brooks, of St. Elmo, Ill.; had suffered for some weeks from symptoms pointing to cancer of the sigmoid, but would not consent to surgical treatment. For a period of about three weeks the bowel-movements were very meager, and finally ceased altogether. Eight days after total obstruction he consented to operation for relief of the enormous distension. On a Saturday perforation of the gut occurred, followed by escape of gas into the free peritoneal cavity, and temporary relief. Remarkable to relate, there was no fever following the comparatively light shock accompanying the emptying of the gut-contents into the peritoneal space, and at no time was the pain excessive. Distension, however, grew more and more distressing, and at time of operation on Wednesday (more than four days after perforation) he seemed to be at the bursting point. His temperature on the fourth day rose to 100.

Opening of the peritoneum in the mid-line was accompanied by the escape of an immense quantity of intestinal gas and a large amount of fecal matter, which was found to be pouring out of a large hole in the descending colon above the point of obstruction. Strange to say, there was not the slightest sign of general peritonitis nor was there any attempt on the part of Nature to repair the injury—neither omentum nor coils of intestine had become glued to the point of perforation.

The belly was irrigated, the colon drawn into the abdominal incision and sutured so as to allow the gut to discharge its contents into gauze at a safe distance from the wound, and gauze drainage inserted in four directions. He lived until the following Friday, and died of general exhaustion—without peritonitis.

The second case was that of I. F., of Maplewood, Mo., patient of Dr. W. H. Townsend, a girl of 16 years, who attended school on Friday, but complained of "cramps" (presumed to be menstrual) that night, increasing to such degree as to require the services of the doctor Saturday. He recognized appendicitis, but the family was not ready for operation. Sunday morning her temperature had risen to 102½, pain was excessive, and abdominal distension intense. Recognizing the fact that this was no ordinary case of appendicitis, Dr. Townsend insisted upon instant operation. About forty-eight hours from the onset the abdomen was opened in the region of the cecum, and was instantly followed by discharge of a large quantity of of liquid feces. Investigation showed the pelvis to be filled with feces and thin pus, all of the fecal stream having passed through a very large opening at the root of the appendix. There was not a sign of agglutination of coils of intestines or omentum—nothing to protect the peritoneum from fecal contamination.

The pelvis was mopped dry, the gangrenous appendix removed, the hole in the gut closed by two rows of sutures, the omentum pulled down around the affected area and gauze-drain inserted. Drainage was very copious for some twelve hours, temperature dropped to normal, and the patient made a perfect recovery without a single unfavorable symptom.

Lesson: One can not always tell what is going on in the peritoneal cavity. Nature sometimes protects; often the surgeon does better.

